# Confirmation of Fusarium root rot resistance QTL *Fsp-Ps 2.1* of pea under controlled conditions

**DOI:** 10.1186/s12870-019-1699-9

**Published:** 2019-03-12

**Authors:** Clarice J. Coyne, Lyndon D. Porter, Gilles Boutet, Yu Ma, Rebecca J. McGee, Angélique Lesné, Alain Baranger, Marie-Laure Pilet-Nayel

**Affiliations:** 1 USDA-ARS Plant Germplasm Introduction & Testing Research, Washington State University, Pullman, WA 99164 USA; 2 USDA-ARS Grain Legume Genetics & Physiology Research, 24106 N. Bunn Road, Prosser, WA 99350 USA; 3 Institut de Génétique, Environnement et Protection des Plantes, INRA, Agrocampus Ouest, Université Rennes 1, 35650 Le Rheu, France; 4 Department of Horticulture, Washington State University, Pullman, WA 99164 USA; 5 USDA-ARS, Grain Legume Genetics & Physiology Research, Pullman, WA 99164 USA

**Keywords:** *Pisum sativum* L., Quantitative trait loci, *Fusarium solani fsp. pisi*

## Abstract

**Background:**

Dry pea production has increased substantially in North America over the last few decades. With this expansion, significant yield losses have been attributed to an escalation in Fusarium root rots in pea fields. Among the most significant rot rotting pathogenic fungal species, *Fusarium solani fsp. pisi* (*Fsp*) is one of the main causal agents of root rot of pea. High levels of partial resistance to *Fsp* has been identified in plant genetic resources. Genetic resistance offers one of the best solutions to control this root rotting fungus. A recombinant inbred population segregating for high levels of partial resistance, previously single nucleotide polymorphism (SNP) genotyped using genotyping-by-sequencing, was phenotyped for disease reaction in replicated and repeated greenhouse trials. Composite interval mapping was deployed to identify resistance-associated quantitative trait loci (QTL).

**Results:**

Three QTL were identified using three disease reaction criteria: root disease severity, ratios of diseased vs. healthy shoot heights and dry plant weights under controlled conditions using pure cultures of *Fusarium solani fsp. pisi*. One QTL *Fsp-Ps 2.1* explains 44.4–53.4% of the variance with a narrow confidence interval of 1.2 cM. The second and third QTL *Fsp-Ps3.2* and *Fsp-Ps3.3* are closely linked and explain only 3.6–4.6% of the variance. All of the alleles are contributed by the resistant parent PI 180693.

**Conclusion:**

With the confirmation of *Fsp-Ps 2.1* now in two RIL populations, SNPs associated with this region make a good target for marker-assisted selection in pea breeding programs to obtain high levels of partial resistance to Fusarium root rot caused by *Fusarium solani fsp. pisi*.

**Electronic supplementary material:**

The online version of this article (10.1186/s12870-019-1699-9) contains supplementary material, which is available to authorized users.

## Background

Dry pea (*Pisum sativum* L.) production has increased substantially in the US since 2004 to 0.5 million hectares, primarily in the northern tier states of North Dakota and Montana [1] and in Canada since the turn of the century to 1.7 million hectares in 2016 [2]. The increase of dry pea cultivation in short rotation with cereal crops has been associated with an increase in root rot incidence in in North America [3, 4], Europe [5, 6] and New Zealand [7].

Root rots impose an important biotic stress on pea production world-wide [8]. Pathogens associated with the root disease complex of pea, recently reviewed in Tran et al. [9], include *Aphanomyces euteiches*, *Fusarium* species, *Phoma pinodella*, *Didymella pinodes*, *Pythium* spp., *Thielaviopsis basicola* and *Rhizoctonia solani*. Kerr [10] was first to note the complexity of root pathogens in the U.S. on pea, albeit with a shorter list of four taxa. Of note, the Fusarium species pathogenic on pea are particularly extensive containing 12 species [11]. *F. solani* f. sp. *pisi* (*Fsp*), *F. acuminatum*, *F. avenaceum*, *F. culmorum*, *F. graminearum*, *F. sambucinum*, *F. equiseti*, *F. oxysporum*, *F. poae*, *F. redolens*, *F. sporotrichioides* and *F. tabacinum* were found pathogenic to varying degrees on pea [5, 11]. In North America, two species have been identified as the most serious production constraints, *Fsp* and *F. avenaceum* [3, 5, 11–13] as determined by field surveys, virulence tests and accompanying confirmation of the species using molecular tools [3, 14]. *F. avenaceum* is the dominant species under reduced or no-tillage practices as it survives on the preceding crop residue [4, 5] and dominates in the northern plains of North America [3, 12]. However, the importance of both species varies by year [12]. *Fsp* is the dominant pathogen in the Pacific Northwest corresponding with mostly conventional tillage used in pea production [13, 15].

*Fsp* has been a troublesome pathogen in pea production since its first report in the U.S. in 1918 [16]. Immunity is unknown [13, 17–19] and the quantitative nature of partial resistance is well described [20, 21]. Genetic resistance to Fusarium root rot (FRR) is a very promising solution as many genetic resources with high levels of partial resistance to *Fsp* are available for breeding [13, 17–19]. However, the genetics of the quantitative partial resistance is little studied with just two QTL reports published for *Fsp* [22, 23].

A field study first identified one QTL for *Fsp* [23]. Based on parental lines with multiple root rot resistances released by Kraft [24], recombinant inbred line populations were developed to study pea root rot resistances with the ultimate goal of developing useful markers for molecular breeding programs [6, 25]. Using one of these RIL populations (DSP × 90–2131), five QTL were identified for *Fsp* resistance using a linkage map based primarily on SSRs associated with the phenotypic disease expression of lines to *Fsp* over 3 years of field evaluations in a FRR nursery in Prosser, WA [22]. One QTL identified, *Fsp-Ps7.1*, was in common with Feng et al. [23]. Field disease nurseries have the confounding factor of potentially other root pathogens being present in the soil and the field disease pressure may be variable across the landscape, which is challenging to control experimentally. The objective of this study was to determine QTL associated with high levels of partial resistance to *Fsp* in a second RIL population (Baccara × PI 180693) under controlled conditions using a higher density SNP-based linkage map called “BP-Duarte” [26].

## Methods

### Plant material

The mapping population was 178 F_8_-derived recombinant inbred lines from the cross of the cultivar ‘Baccara’ (susceptible) and PI 180693 (partially resistant) [25]. Baccara is a semi-dwarf, semi-leafless (afila) dry pea cultivar with a clear seed coat, round seeds and white flowers (Florimond-Desprez, France, registered in 1992). PI 180693, a cultivar ‘Hohenheimer Pink-Flowered’ from Germany [27] is one of the multiple-pathogen resistant parents also used by Kraft [24] to develop multiple root disease resistance germplasm and is tall, with normal leaves, pigmented seed coat, round seed and pink flowers.

### Greenhouse phenotyping

The screening procedure of Bodah et al. [28] was followed using the same mix in each test of an equal mix of three *Fsp* isolates (Fs 02, Fs 07 and Fs 09) isolated from infected pea roots collected in the Palouse Region of Washington and Idaho, USA. Briefly, the single-spored isolates were identified as *Fsp* using partial translation elongation factor 1-*a* sequences [14]. Isolates were grown for inoculum production on pentachloronitrobenzene (PCNB) [29] agar then transferred to Kerr’s media [10] and incubated six days. Spores were collected by centrifugation and resuspended in sterile deionized water (sdH_2_0) and diluted to 1 × 10^6^ spores/ml water. Seeds of each genotype were surface disinfested with a 10% bleach solution, rinsed in sdH_2_O and soaked for 16 h in either the spore suspension of *Fsp* isolates or in sdH_2_O for the non-inoculated control, then planted in perlite in containers (Conetainer, 0.25 L volume, Stuewe and Sons Inc.). The experimental design was a split-plot design, the main plot (test = 3) was the environment factor, the subplots (*n* = 2) were the lines, with five or six randomized single plant replicates per line per test. Three tests were used in this study. Both Test 1 and 2 included the whole population, 178 lines and Test 3 included 89 lines. Plants were irrigated as needed, a 12-h photoperiod was maintained and greenhouse temperatures were set at 29.4°C during the day and 23.9°C at night.

The disease evaluation values were taken similar to Bodah et al. [28]. Root disease severity (RDS), plant height, and shoot dry weights were recorded 25 days after planting. RDS was assigned based on a visual scale from 0 to 6 adapted from Hance et al. [30]; where: 0 = no diseases symptoms; 1 = small hypocotyl lesions; 2 = lesions coalescing around epicotyls and hypocotyls; 3 = lesions starting to spread into the root system with some root tips infected; 4 = epicotyl, hypocotyl and root system almost completely infected and limited white, uninfected tissue visible; 5 = completely infected root; and 6 = plant failed to emerge. Each genotype was evaluated in two to three separate tests (Additional file 1). Scores of two surrogate traits were recorded of shoot dry weights (weight loss) and plant heights (height loss) between inoculated plants and non-inoculated plants similar to Bodah et al. [28] where 1 = greater than 100% normal growth; 2 = 76 to 100% of normal growth; 3 = 51 to 75% of normal growth; 4 = 26 to 50% of normal growth and 5 = 0 to 25% of normal growth.

### Phenotypic data analysis

Data was analyzed as three tests with two methods. First, ordinal logistic regression, a statistical analysis method that can be used to model the relationship between an ordinal response variable and one or more explanatory variables [31]. An ordinal variable is a categorical variable for which there is a clear ordering of the category levels. Ordinal logistic regression is an extension of logistic regression where the logit (i.e. the log odds) of a binary response is linearly related to the independent variables. The output of an ordinal logistic regression will contain an intercept for each level of the response except one, and a single slope for each explanatory variable pooled across tests for RDS, plant weight and plant height loss. Ordinal logistic regression was applied with fixed effects for lines and environments using R [32] (Additional file 2). The trait data were also analyzed by analysis of variance (ANOVA) using the mixed linear model (MIXED) procedure with genotype as fixed effect and environment as random effect in SAS University Edition [33]. The statistical model is *Y*_*ij*_ *= β*_*0*_ *+ β*_*1*_*x*_*i*_ *+ u*_*1*_*z*_*j*_ *+ ε*_*ij*_, where *Y*_*ij*_ is the response variable, *β*_*0*_ is the overall mean, *β*_*1*_ is the genotype effect, *u*_*1*_ is environmental effect, and *ε*_*ij*_ is the effect from errors terms. The broad-sense heritability (*H*^*2*^) was calculated for each trait as *H* ^*2*^ *=* σ_G_^2^/[σ_G_^2^ + (σ_GE_^2^/*e*) + σ_e_^2^/*re*], where σ_G_^2^ = genotypic variance, σ_GE_^2^ = variance due to interaction between genotype and environment, σ_e_^2^ = error variance, *e* = number of environments, *r* = number of replicates. The variance components were calculated by SAS PROC MIXED with all the effects considered as random effects. PROC UNIVARIATE option of SAS was used to generate summary statistics including skewness and kurtosis [33]. Pearson correlation coefficients among the traits were calculated using SAS PROC CORR.

QTL analysis.

The BP-Duarte linkage map [26] was comprised of 914 markers covering 1073 cM on seven linkage groups (LG) and used genotyping data obtained from SSRs [25] and cDNA derived SNPs [34] markers on ‘Baccara’ x ‘PI180693’ 178 RIL population. The Baccara x PI180693 map was from one of the four RIL populations used to build the composite genetic map described in Duarte et al. [34]. The linkage map included 701 SNPs identified from transcriptome sequencing and derived from a Goldengate assay [34], as well as 179 SSRs and 35 other markers (RAPDs, specific PCRs, morphological genes) used in the initial version of the genetic map established from this population [25]. Composite interval mapping (CIM) implemented in QTLCartographer 2.5 software was used to identify significant QTL using mean scores for root disease severity, disease plant height (control vs inoculated), and disease plant weight (control vs inoculated) [35]. Significant LOD threshold was set to 2.8 using Doerge and Churchill’s [36] test of 1000 permutations before CIM. Settings for CIM included QTLCartographer Model 6 (default) except for selection of a walk speed of 2 cM and the forward and backward regression method (*P* <  0.1). QTL were declared exceeding the LOD threshold and defined with the range of ±1 LOD (95% confidence interval). MapChart was used to draw the linkage map and QTL locations [37].

## Results

### RIL population root rot evaluation

**Fig. 1:**
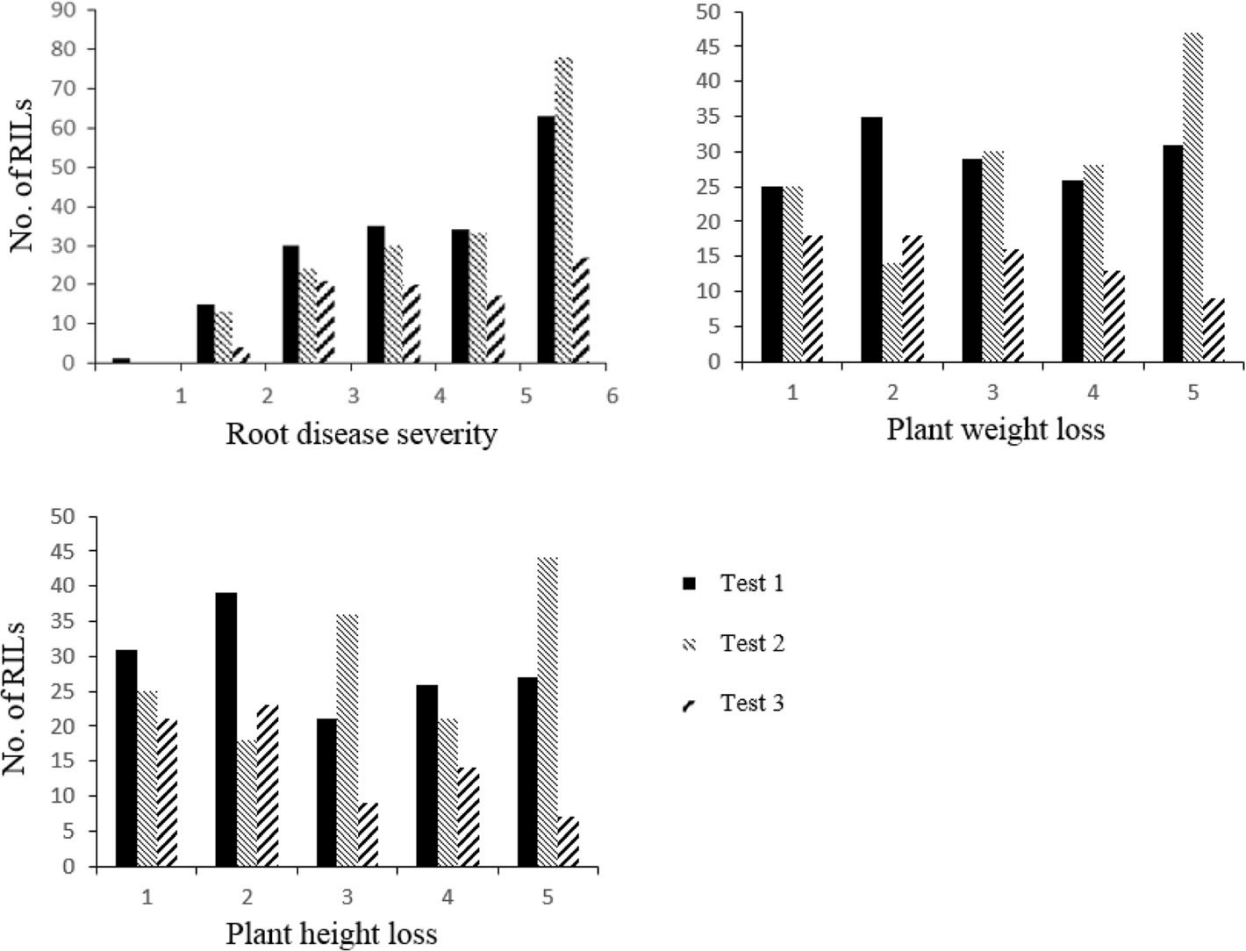
Frequency histograms of disease severity, plant weight loss and plant height loss scores

The RIL population of 178 lines was evaluated for reaction to three isolates of *Fsp* under controlled conditions and displayed transgressive segregation for both increased susceptibility and increased resistance over the two parental lines as measured by the three disease response traits (Fig. 1, Table 1). The parental reaction fit the expected response for disease severity and plant weight loss, but the parents did not differ for plant height loss (Table 1). Moderate skewness and kurtosis were noted for the three disease scores. Mean disease ratings are skewed towards susceptibility (Fig. 1). This is due to the non-emergence of treated seed where those susceptible lines receive a disease severity score of 6, plant weight loss and height loss are scored 5. Using ANOVA, the RILs were significantly different in all the traits (Table 2). In terms of environmental effects, there were no significant differences in all traits (*P*_disease severity_ = 0.19, *P*_plant weight_ = 0.21, and *P*_plant height_ = 0.20) using the mixed linear model frequently employed in QTL studies. The environment (test) was significant using ordinal logistic regression for RDS and plant weight loss (Additional file 2). Genotype by environment interaction also had a significant effect (Table 2). All three traits are highly and significantly correlated (Table 3). Broad-sense heritability was high for root disease severity but on the low side for plant height loss and plant weight loss (Table 1) reducing their value as surrogate traits.

**Table 1:** Statistical summary of the traits for the parents, the RILs and calculated broad-sense heritabilities based on a mixed linear model

	PI 180693	Baccara	RILs			
	Mean	Mean	Mean	CV	Range	*H* ^*2*^
Root disease severity	2.7	4.4	4.2	47.0	0–6	78.8%
Plant weight loss	1.4	3	3.1	46.3	1–5	46.2%
Plant height loss	2.4	2.6	3.0	49.1	1–5	43.4%

**Table 2:** ANOVA based on the mixed linear model for root disease severity, plant weight loss and plant height loss scores of the RIL population

	Source	Z Value	Pr > Z
Root disease severity	Genotype	5.62	< 0.01
	Environment (test)	0.87	0.19
	Genotype × Environment	4.12	< 0.01
Plant weight loss	Genotype	1.87	< 0.01
	Environment (test)	0.82	0.21
	Genotype × Environment	5.95	< 0.01
Plant height loss	Genotype	1.78	< 0.01
	Environment (test)	0.84	0.20
	Genotype × Environment	6.03	< 0.01

**Table 3:** Pearson correlation coefficients between the three traits measured for reaction to both inoculation and water-treated control plants of disease symptoms per se, percentage plant heights and percentage dry weights converted to a 0–6 scale (disease severity) and 1–5 scale (plant height loss and weight loss scores)

	Root disease severity	Plant weight loss	Plant height loss
Root disease severity	1.00	0.79^a^	0.78^a^
Plant weight loss		1.00	0.93^a^
Plant height loss			1.00

^a^Significant at the 0.001 probability level

### QTL for root rot resistance

Three significant QTL were identified for partial resistance to FRR using the three traits measured with the three alleles from the resistant parent PI 180693 (Table 4). *Fsp-Ps2.1* is the strongest QTL with high LOD peaks (25.3–32.4) explaining 44.4 to 53.4% of the variance for resistance and identified with all three traits. The position of the QTL is the same for RDS and the surrogate traits weight/height loss and the LOD-1 interval is relatively small (1.2 cM). The second and third QTL *Fsp-Ps3.2* and *Fsp-Ps3.3* are closely linked, their LOD peaks are just above the LOD cutoff of 2.8, explain much less of the variance, 3.6 to 4.6% and the allele effects are small (Additional file 3). SNP marker detail for each QTL identified is included in Additional file 3. A figure of the linkage groups of non-redundant markers with QTL locations and the LOD intervals (LOD peak ±1 and ± 2) is presented in Additional file 4.

**Table 4:** Quantitative trait loci detected for resistance to Fusarium root rot in Baccara × PI 180693 recombinant inbred lines population using RDS, weight loss and height loss scores

LG^a^	QTL name	Scoring trait	Position (cM)	Closest left marker from the position	LOD^b^ peak	LOD-1 support interval (cM)	R^2^ (%)	Additive effect^c^
II	*Fsp-Ps2.1*	Root disease severity	49.3	Ps900203	32.4	1.2	53.4	0.92
	*Fsp-Ps2.1*	Height loss	49.3	Ps900203	25.3	1.2	44.4	0.80
	*Fsp-Ps2.1*	Weight loss	49.3	Ps900203	27.9	1.2	50.5	0.83
III	*Fsp-Ps3.2*	Root disease severity	23.5	Ps900299	3.14	23.0	3.9	0.24
	*Fsp-Ps3.3*	Height loss	35.3	Ps900382	3.24	16.8	4.6	0.27
	*Fsp-Ps3.3*	Weight loss	35.3	Ps900195	2.94	25.2	3.6	0.22

^a^Pea linkage group as assigned in [25]

^b^Logarithm of odds

^c^Effect of substituting Baccara alleles for PI 180693 alleles at the QTL. A positive sign indicates that QTL alleles increasing the resistance are contributed by the resistant parent PI 180693, whereas a negative sign means resistant alleles are contributed by the susceptible parent Baccara

## Discussion

Denser pea linkage maps using genotyping-by-sequencing technologies have enabled localizing significant disease resistance QTL to smaller regions giving the prospect of fine mapping and eventual cloning of the underlying resistance genes higher probabilities (e.g. [38]; review [39]). Prior to determining the causal gene(s), the use of SNPs allows the development of breeder friendly markers using marker systems such as KASP [40]. Here we report a significant QTL *Fsp-Ps2.1* that explains up to 53% of the variance and has a small confidence interval. This QTL can be used in plant breeding programs to increase the level of partial resistance to *Fsp*. We hypothesize the QTL *Fsp-Ps2.1* is the same as reported from a field study of another RIL population [22] and we assigned the same name in this report. This is based on comparative mapping between the two RIL populations using common SSR markers, a common parent (PI 180693) in the pedigree of 90–2131, and the similar high variance of resistance explained. Finer mapping in the previous report’s RIL population is necessary to confirm this hypothesis.

In this report’s *AA* × *aa* RIL population, *A* (pigmented flower/anthocyanin pigmentation) maps with the interval of *Fsp-Ps2.1* and thus is a potential candidate gene. In Coyne et al. [22] *Fsp-Ps2.1* was mapped in the white flower (*aa* x *aa*) cross of DSP × 90–2131 so the location of *A* was estimated to be distal to one SSR marker. One hypothesis is that the resistance gene(s) responsible for *Fsp-Ps2.1* effect may not necessarily be *A* since *Fsp-Ps2.1* was originally identified in that white (*a*) flowered cross. The white flowered, resistant parent of this RIL population, 90–2131, was derived from a complex cross in which PI 180693 (AA) was the male parent and is resistant to FRR [24]. Kraft may have successfully broken the linkage between *Fsp-Ps2.1* and *A* in white-flowered *Fsp* resistant lines he released [24, 41, 42]. Alternatively, a different gene(s) for FRR resistance was transferred in those crosses [24, 41, 42]. Fine mapping in both populations will be necessary to test this hypothesis.

The second QTL, *Fsp-Ps3.2*, is new, distal to the previously reported QTL on linkage group III, *Fsp-Ps3.1* [22]. *Fsp-Ps3.2* and *Fsp-Ps3.3* may harbor weaker resistant alleles as they have low LOD scores and explain less of the variance (up to 4.6%) than *Fsp-Ps3.1* (up to 9.9%). *Fsp-Ps3.2* or *Fsp-Ps3.3* may possibly be one of the same QTL for *Fsp* near the *M* gene (brown mottling of testa) reported in [30]. However, we were unable to map the *M* locus as it is not segregating in this report’s population to confirm this possibility. *Fsp-Ps3.2* may be of more interest for resistance as it was detected with RDS score while *Fsp-Ps3.3* may be more linked to tolerance as it was only detected with the surrogate traits of plant weight and height loss. The confidence intervals for both overlap and are large, up to 25.2 cM so it would be more difficult to use in breeding for resistance to *Fsp*.

Screening larger RIL populations for plant disease resistance in greenhouses necessitates large numbers of replicates and untreated check lines. Physical space limitations and phenotyping capability constraints dictate compromises on the experimental design. The analysis of the ordinal data using ordinal logistic regression was effective for the data analysis. It revealed that the split plot design with line as the subplot was significant so was not the optimum choice (Additional file 2). A better design would be a randomized complete block design with fewer replicates per test when confronted with space limitations. Additionally, to overcome the challenge of limited data collection capability, high-throughput phenotyping platforms may relieve the bottleneck and enable faster and more efficient phenotypic data collection.

As noted in Coyne et al. [22], *Fsp-Ps2.1* collocated with the Aphanomyces root rot partial resistance QTL *Ae-Ps2.1* based on sparse common SSR markers from Hamon et al. [25]. Now, using a denser SNP-based linkage map, *Fsp-Ps2.1* clearly collocates with an adjacent QTL, *Ae-Ps2.2* [25]. QTL meta-analysis identified MQTL-Ae5/Ae6 for Aphanomyces at this location provides evidence for the importance of this allele for root rot resistance in pea [43]. Additionally, *Fsp-Ps3.2* collocates with Aphanomyces root rot partial resistance QTL *Ae-Ps3.1*.

Interestingly, strong QTL controlling partial resistance to Fusarium root rot have been reported in other legumes, among which one presented a high broad sense heritability. In soybean, one QTL for resistance to *Fusarium graminearum* explained 38.5% (LOD 23.9) of the phenotypic variance with high *H*^*2*^ of 0.79 [44] similar to *Fsp-Ps2.1* and our calculated *H*^*2*^. A second soybean publication identified a QTL explaining 40.2% of the variance (LOD 20.3) for resistance to *F. graminearum* [45]. In a common bean study, an important QTL explained 23% of the variance (LOD 11.5) and a second one with lower percentage of the variance (9%) was reported [46]. However, narrow sense heritabilites were very low (*h*^*2*^ = 0.19–0.20) [46]. In a second bean study, one QTL was identified for FRR explaining just 10% of the variance (LOD 3.2) [47]. Conversely, in a recent bean study, QTLs for *F. solani* resistance identified 17 QTL related to FRR explaining at most 16% of the variance (LOD 5.84) [48]. In the closely related legume, lentil, Fusarium root rot was identified as a problem by Hwang et al. [49] and noted as an increasing production constraint [4]. However, no genetic studies on resistance to FRR in lentil have been published to date.

None of the legume root rot resistance genes are known to date (review [39]), however an F-box encoding gene is strongly suggested to be implicated for Aphanomyces resistance in *Medicago truncatula* [50]. The resistance gene to *F. graminearum* in a non-legume crop has been identified in wheat as a chimeric lectin for head blight but not crown rot resistance[51]. *Fsp-Ps2.1* may be an interesting target for determining the gene(s) most responsible for the high levels of partial resistance found in both RILs reported here and in Coyne et al. [22]. Interestingly, using a transgenics approach in pea, four antifungal genes *1–3 β glucanase* (G), *endochitinase* (C) (belonging to PR proteins family), *polygalacturonase inhibiting proteins* PGIPs) (P) and *stilbene synthase* (V) did not consistently improve disease resistance to *F. avenaceum* in field studies [52] so perhaps *Fsp-Ps2.1* is not any of these genes. New genomic tools, innovations in phenotyping and VIGs technology in pea will assist in the determination gene(s) for high levels of partial resistance to *Fsp* [53–55].

## Conclusions

The confirmation of the important QTL *Fsp-Ps2.1* is a step forward in understanding high levels of partial resistance to Fusarium root rot, caused by *Fsp,* in pea. The high level of the variance explained and the small confidence interval indicate the SNPs associated with this QTL are a good target for marker assisted breeding. The interval for QTL *Fsp-Ps3.2* is too large and would need additional fine mapping to provide informative markers.

## Additional files

Additional file 1: Phenotypic data for the disease severity, plant height loss and plant weight loss scores of the RIL population. (XLSX 69 kb)

Additional file 2: R code for ordinal logistic regression and ANOVA. (DOCX 16 kb)

Additional file 3: SNP polymorphism and adjacent sequence of QTL-associated markers reported for disease severity in Table 4. (XLSX 12 kb)

Additional file 4: Linkage map and QTLs. Black box ±1 LOD and line bars ±2 LOD from peak. (PDF 719 kb)

## Abbreviations

CIM: composite interval mapping
*Fsp*: *Fusarium solani fsp. Pisi*
QTL: quantitative trait locus
RDS: root disease severity
SNP: single nucleotide polymorphism
SSR: simple sequence repeat
