# Supplementary material for: Confirmation of Fusarium root rot resistance QTL Fsp-Ps 2.1 of pea under controlled conditions
Source: BMC Plant Biol. 2019 Mar 12;19:98. doi: 10.1186/s12870-019-1699-9 (PMC6417171; doi:10.1186/s12870-019-1699-9)
Supplement: Supplementary file 2 — R code for ordinal logistic regression and ANOVA. (DOCX 16 kb) [file 12870_2019_1699_MOESM2_ESM.docx]

Additional file 2: R code for ordinal logistic regression and ANOVA.

***R code***

library("MASS")

library("nlme")

####common varieties - diseas severity####

mydata=read.table(file = "C:/Users/Yu/Desktop/CISER/project4_common_varieties_disease_2.txt",header = TRUE,na.strings = ".")

mydata[1:10,1:4]

mydata$Disease=as.factor(mydata$Disease)

cv1 <- polr(Disease~Test+Line+Rep, data=mydata)

cv2 <- polr(Disease~Rep+Line, data=mydata)

anova(cv1,cv2)

####whole data set - disease severity####

mydata_whole=read.table(file = "C:/Users/Yu/Desktop/CISER/Project4_wholedataset_disease_2.txt",na.strings=".",header = TRUE)

mydata_whole$Line=as.factor(mydata_whole$Line)

mydata_whole$Disease=as.factor(mydata_whole$Disease)

analysis1=polr(Disease~Line+Rep+Test, data=mydata_whole)

analysis2=polr(Disease~Line+Test, data=mydata_whole)

anova(analysis1,analysis2)

analysis3=polr(Disease~Line+Rep, data=mydata_whole)

anova(analysis1,analysis3)

analysis4=polr(Disease~Rep+Test, data=mydata_whole)

anova(analysis1,analysis4)

####common varieties - disease weight####

mydata=read.table(file = "C:/Users/Yu/Desktop/CISER/project4_common_varieties_weight.txt",header = TRUE,na.strings = ".")

mydata[1:5,1:3]

mydata$Weight=as.factor(mydata$Weight)

cv1 <- polr(Weight~Test+Line, data=mydata)

cv2 <- polr(Weight~Line, data=mydata)

anova(cv1,cv2)

####whole data set weight####

mydata_whole=read.table(file = "C:/Users/Yu/Desktop/CISER/project4_wholedataset_weight_3.txt",header = TRUE,na.strings = ".")

mydata_whole$Weight=as.factor(mydata_whole$Weight)

mydata_whole$Test=as.factor(mydata_whole$Test)

analysis1=polr(Weight ~Line+Test, data=mydata_whole)

analysis2=polr(Weight ~Test, data=mydata_whole)

anova(analysis1,analysis2)

analysis3=polr(Weight ~Line, data=mydata_whole)

anova(analysis1,analysis3)

####common varieties -disease height####

mydata=read.table(file = "C:/Users/Yu/Desktop/CISER/project4_common_varieties_height.txt",header = TRUE,na.strings = ".")

mydata[1:5,1:3]

mydata$Height=as.factor(mydata$Height)

cv1 <- polr(Height~Test+Line, data=mydata)

cv2 <- polr(Height~Line, data=mydata)

anova(cv1,cv2)

####whole data set - disease height####

mydata_whole=read.table(file = "C:/Users/Yu/Desktop/CISER/project4_wholedataset_height_nomissing.txt",header = TRUE,na.strings = ".")

mydata_whole[1:5,1:3]

mydata_whole$Height=as.factor(mydata_whole$Height)

analysis1=polr(Height ~Line+Test, data=mydata_whole)

analysis2=polr(Height ~Line, data=mydata_whole)

anova(analysis1,analysis2)

analysis3=polr(Height ~Test, data=mydata_whole)

anova(analysis1,analysis3)

ANOVA-Ordinal logistic regression-R

|  | Source | P value | S/NS |
| --- | --- | --- | --- |
| Disease severity | Genotype | 0 | S |
|  | Environment | 3.7e-13 | S |
| Disease weight score | Genotype | 3.7e-10 | S |
|  | Environment | 0.01 | S |
| Disease height score | Genotype | 2.3e-11 | S |
|  | Environment | 0.35 | NS |
