# Supplementary figures and images for: Confirmation of Fusarium root rot resistance QTL Fsp-Ps 2.1 of pea under controlled conditions
Source: BMC Plant Biol. 2019 Mar 12;19:98. doi: 10.1186/s12870-019-1699-9 (PMC6417171; doi:10.1186/s12870-019-1699-9)

LGII\_BP-Duarte

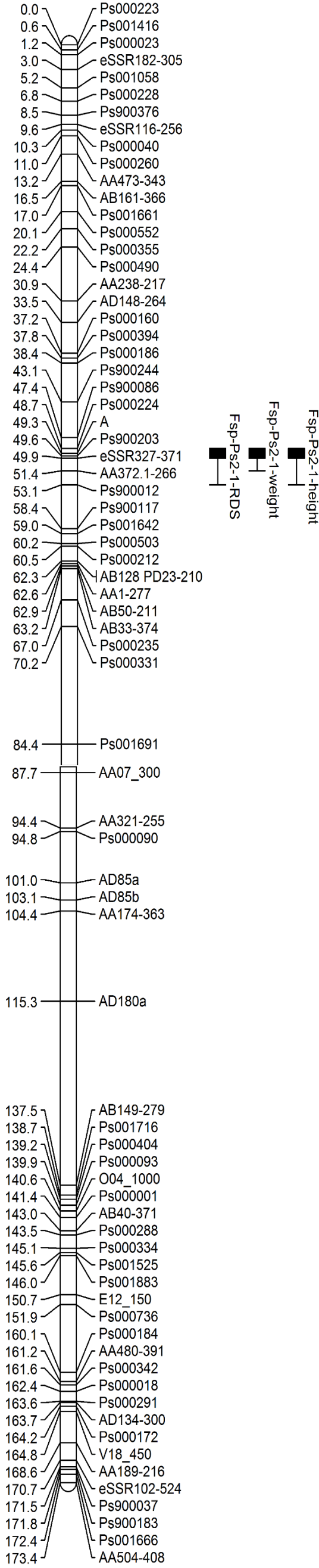

LGIII\_BP-Duarte

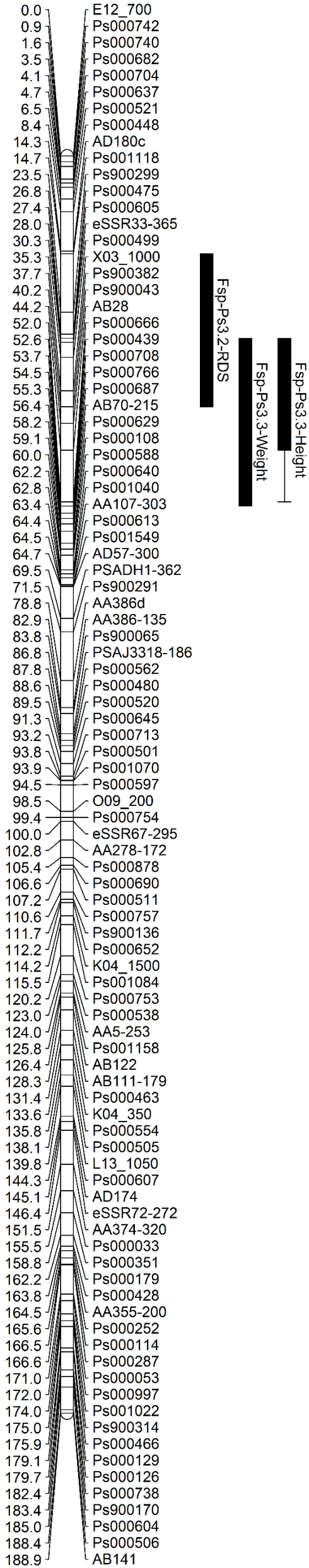

Supplement: Supplementary file 4 — Linkage map and QTLs. Black box ±1 LOD and line bars ±2 LOD from peak. (PDF 719 kb) [file 12870_2019_1699_MOESM4_ESM.pdf]
